# Predictors of *Plasmodium falciparum* Malaria Incidence in Chano Mille, South Ethiopia: A Longitudinal Study

**DOI:** 10.4269/ajtmh.2012.12-0155

**Published:** 2012-09-05

**Authors:** Eskindir Loha, Bernt Lindtjørn

**Affiliations:** School of Public and Environmental Health, Hawassa University, Ethiopia; Centre for International Health, University of Bergen, Norway

## Abstract

We assessed potential effects of local meteorological and environmental conditions, indoor residual spraying with insecticides, insecticide-treated nets (ITNs) use at individual and community levels, and individual factors on *Plasmodium falciparum* malaria incidence in a village in south Ethiopia. A cohort of 8,121 people was followed for 101 weeks with active and passive surveillance. Among 317 microscopically confirmed *P. falciparum* malaria episodes, 29.3% occurred among temporary residents. The incidence density was 3.6/10,000 person-weeks of observation. We observed higher malaria incidence among males, children 5–14 years of age, ITNs non-users, the poor, and people who lived closer to vector breeding places. Rainfall increased and indoor residual spraying with Deltamethrin reduced falciparum incidence. Although ITNs prevented falciparum malaria for the users, we did not find that free mass ITNs distribution reduced falciparum malaria on a village level.

## Introduction

Malaria is a major public health problem in Ethiopia, and in 2011 about two-thirds of the population was at risk of malaria.^1^ Results of the 2007 national malaria indicator survey showed prevalence of 0.7% and 0.3% of *Plasmodium falciparum* and *Plasmodium vivax* malaria, respectively^2^; the dominant vector is *Anopheles arabiensis*. Indoor residual spraying (IRS) with insecticides has been used since 1960, and distribution of insecticide-treated nets (ITNs) to all age groups and provision of free artemisinin-based combination therapy were in use since 2004.^1^

In the southern part of the country, malaria remains the leading cause of outpatient morbidity, admission, and death comprising 27.6%, 28.7%, and 46.4%, respectively, in the year 2009/2010.^3^ In 2010/2011, 1,360,101 individuals received antimalarial drugs, and 490,729 were laboratory-confirmed cases. In the same year, IRS with Deltamethrin was carried out in 1,129,158 houses covering a total of 3,850,808 residents, and 525,177 ITNs were distributed to 262,589 households. The estimated coverage of ITNs was 95%,^4^ and this was greater than the national average of 72%.^5^ Despite these interventions, a more than expected number of malaria cases was reported from some areas, but no mention was made on the possible reasons for the increase.^4^

Understanding the multifaceted determinants of malaria transmission is important. Many factors play a role in malaria transmission including local meteorological conditions,^6^,^7^ population movement,^8^,^9^ age, sex, socio-economic factors,^10^–^15^ and prevention and control measures.^16^–^18^

Some studies showed that IRS and ITNs prevent and control malaria transmission using a varying scale of analyses for model, time, and space.^16^–^19^ The simultaneous use of IRS and ITNs might be beneficial.^20^,^21^ However, rising insecticide resistance of vectors could hamper the effectiveness of such insecticide-based interventions.^22^ In addition, a high percentage of ITNs ownership may not be a guarantee for a sustained lower incidence of the disease caused by factors possibly linked to lack of efficacy or lack of proper use.^23^–^26^

Although a comprehensive analytical approach to understanding malaria epidemiology may not be possible, investigating the roles of as many factors as possible might shed light on prevention and control strategies. Therefore, our study aimed to assess the effect of local meteorological and environmental conditions, IRS, ITNs use at individual and community levels, and socio-economic and other individual-level factors on falciparum malaria incidence in a village in south Ethiopia.

## Methods

### Study setting.

**Figure 1.**
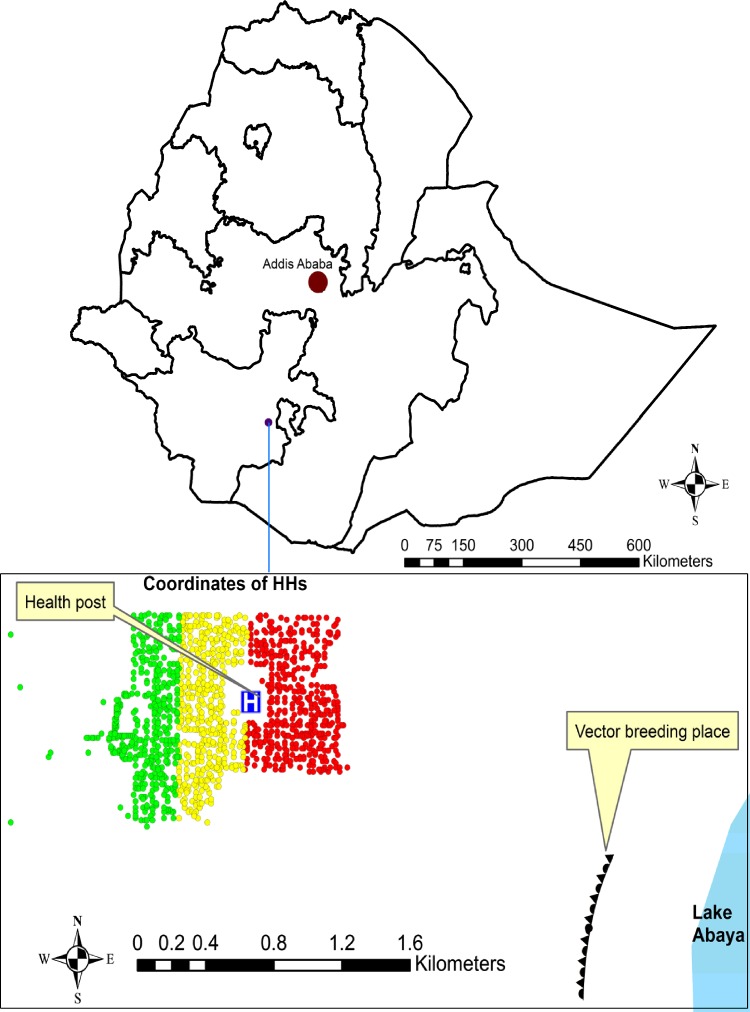
Map of Ethiopia, location of Chano Mille Kebele and coordinates (dots) of households subdivided into sub-Kebeles 1 (green), 2 (yellow), and 3 (red), 2009–2011.

This study was carried out in rural Chano Mille Kebele of Arba Minch Zuria district that is 492 km south of Addis Ababa. The Kebele was selected purposely to study malaria epidemiology and vector biology in detail. Kebele is the smallest administrative region and each Kebele had at least one government health post. A health post provides basic health services including malaria diagnosis using rapid diagnostic test (RDT) kits and treatment with Co-Artem (for falciparum malaria only), which is free. The research project provided Chloroquine tablets (for vivax malaria). The Kebele area was 2.4 sq km, and the health post was located at 6°6.666′ N and 37°35.775′ E. The altitude is 1,206 meters above sea level. Almost all households had mango plantation within their compound. Mango, banana, and maize were the major cash crops. Geographical location and description of the study area is shown in Figure 1.

### Study design.

This cohort study was carried out in collaboration with the village health post. The study covered 101 weeks, from April 2009 to April 2011. All residents in the Kebele were taken as study subjects. Every household was visited weekly looking for febrile cases. A febrile case was a case whose axillary temperature was ≥ 37.5°C. The active surveillance team used to refer a febrile case to the health post for diagnosis and treatment. At the end of each day there was a cross-check whether that case had visited the health post. All households were given a unique identification card with a number corresponding to the unique number posted on a metal plate on the main entrance of each house. Residents were advised to come to the health post with the identification card if they got febrile in the days between the weekly visits (passive surveillance).

### Data.

**Figure 2.**
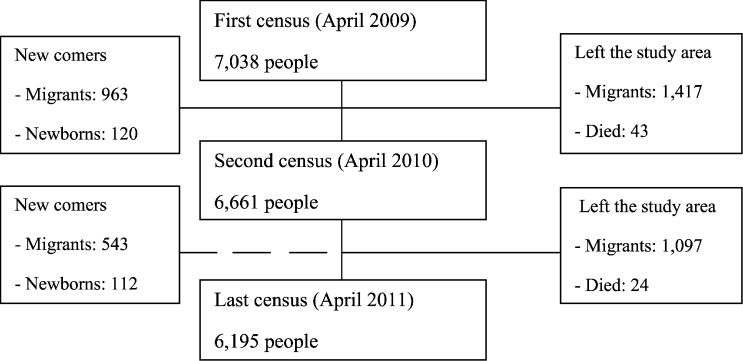
Study profile: broken line indicates that follow-up on insecticide-treated nets (ITNs) use was not done, Chano Mille Kebele, south Ethiopia, 2009–2011.

The geographic coordinates of all houses and vector breeding places were recorded by using a handheld global positioning system receiver with an accuracy of ± 5 m. Census was done at the beginning, on Week 50, and at the end. In the first census, 7,038 (1,212 households) individuals were registered, and the second census added 1,083 subjects—making the total number of studied subjects 8,121 in 1,388 households. The average number of persons per household was 5.9. Figure 2 shows the study profile. The month in which the study subjects came into or left the study area was recorded. The month in which the study subjects left or moved to the study area was considered to contribute 2 weeks to the total number of weeks observed.

Blood samples were collected from all febrile cases using World Health Organization (WHO) standard procedures^27^; a single finger prick was used to take a blood sample for the RDT and to prepare thick and thin blood films for microscopic evaluation. The result of RDT was used to treat the febrile case on the spot. The confirmations of at least two of the three experienced readers were sought to label a study subject as a malaria case. This work dealt with microscopically confirmed (by at least two readers) malaria cases only.

Three major malaria-related interventions (IRS with DDT and later with Deltamethrin and mass ITNs distribution) were carried out by the government within the study period. The brand of the ITNs was PermaNet2.0 (Vestergaard, Frandsen, Switzerland). We did post-intervention surveys to record ITNs and IRS coverage, and document replastering of the insecticide sprayed surfaces in the houses. The efficacies of DDT and Deltamethrin were assumed to be 10% and 50% (taken from Massebo and colleagues, unpublished data of the same study area) on the sprayed week and to decay over time at a constant rate at each time step—becoming nil after 24 weeks and 18 weeks,^21^,^28^ respectively. The initial efficacy was multiplied by the coverage minus the proportion of households that practiced replastering of the sprayed surface.

Starting from Week 5, we recorded each household member who slept under the ITNs the night before the interview.

The main vector breeding place was identified by the research team. We explored potential places within and surrounding the Kebele and the place where we found larvae of *Anopheles* species was the swampy area near the Lake Abaya. There were several small water bodies created mainly by hoof-prints of cattle and hippopotamus. Being on the shore of the lake, these tend to dry slowly after the rainy season—producing extended effect of rainfall. Overflow of the lake during the rainy season followed by shrinkage resulted in more favorable condition for vector breeding.

The meteorological data were obtained from the nearest local meteorological station at Arba Minch University, which was 6 km away from the study area.

### Statistical analysis.

Time-series modeling (auto-regressive integrated moving average [ARIMA] with transfer function [TF]) was carried out considering rainfall, temperature (minimum and maximum), relative humidity, ITNs use fraction, and IRS as potential predictors. The timescale was a week. The ITNs use fraction and IRS was lagged by 2 weeks considering the incubation period of falciparum malaria. The ITNs use data were available from Week 5; therefore, the analysis involved malaria incidence data starting from Week 7. This made the serial length 95 weeks. The number of individuals who slept under the net the night before the interview was divided by the total population of the week as a denominator to get the ITNs use fraction. The total population varied from week to week based on the number of weeks each individual has been observed. Cross-correlations were used to get information about lag number of meteorological variables. The maximum number of lags was set to be 16. Significant peaks (peaks with a bar height beyond the upper or lower confidence limit) in the cross-correlation function plot determined the lag numbers for further analysis. Ljung-Box Q statistics was used as model diagnostics (the *P* value should not be < 0.05 to accept the model). The methodological details of ARIMA and TF models were reported elsewhere.^29^

To construct a wealth index, principal component analysis (PCA) was carried out using 15 socio-economic variables (see Supplemental file for the details). Lessons to construct the wealth index were taken from Vyas and Kumaranayake, and Howe and colleagues.^30^,^31^ The total variance explained by the first principal component and the corresponding Eigen value was 20.35% and 3.05%, respectively, and this was comparable with a previous study.^12^ A factor score derived from the first principal component was used in further analysis as a wealth index. Wealth index categorized into tertiles was used for descriptive purposes.

A generalized Poisson log-linear model was built considering age, sex, wealth index, proximity to the identified vector breeding place, total ITNs use, and education of the head of the household as potential predictors of falciparum malaria episodes for each study subject. The mean and the variance of falciparum episodes were 0.03 and 0.032, respectively. The number of weeks an individual had been observed was considered as a scale weight variable. Pearson χ^2^ was used as scale parameter method and robust estimator for the covariance matrix. The parameter estimation method was hybrid with a maximum Fisher scoring iteration of 1. Kernel was specified for the log-likelihood function. Bivariate and multivariate analyses were carried out. The term used to build the model was the main effects. The omnibus test was used to check performance of each fitted model against the intercept-only model.

Because there was weekly active surveillance for cases, the generalized estimating equation (GEE) was carried out to allow for repeated measurements. The probability distribution specified was binomial with logit link function with independent working correlation matrix. Pearson χ^2^ was used as the scale parameter method and robust estimator for the covariance matrix. The parameter estimation method was hybrid with a maximum Fisher scoring iteration of 1. Kernel was specified for the log quasi-likelihood function. The term used to build the reported model was the main effects. Corrected quasi-likelihood under independence model criterion was compared for main and interaction effects but no model improvement was achieved. The hypothesis test was based on Wald χ^2^. Wealth index, age, sex, proximity to the identified vector breeding place, ITNs use, and education of the head of the household were considered as predictors. Because data on ITNs use at the individual level was collected from Week 5 and net use lagged by 2 weeks; data on ITNs use and falciparum malaria incidence ranged from Weeks 5 to 99 and Weeks 7 to 101, respectively, making the total number of weeks observed 95. The minimum and maximum number of observations per individual was 2 and 95.

Pairwise comparisons of estimated marginal means for age categories were done based on the original scale of dependent variable. The adjustment method for multiple comparisons was Sequential Sidak, which is a sequentially step-down rejective Sidak procedure that is much less conservative in terms of rejecting individual hypotheses but maintains the same overall significance level.

PASW Statistics 18 (Chicago, IL) was used for the analyses. Distance (in meters) from the vector breeding place was calculated using the proximity analysis tool of ESRI ArcMap 9.3(Redlands, CA). To calculate the incidence rate ratio (IRR) with test-based confidence intervals (CI), we used Stata/IC 11.0 (College Station, TX).

### Ethical approval.

The Regional Health Research Ethics Review Committee of the Southern Nations, Nationalities and People's Regional Health Bureau approved this research project. Informed verbal consent was obtained from all study participants. For minors, consent was obtained from their caregivers or legal guardians. Patients were treated according to National guidelines^32^ with antimalarial drugs immediately based on their RDT results.

## Results

**Figure 3.**
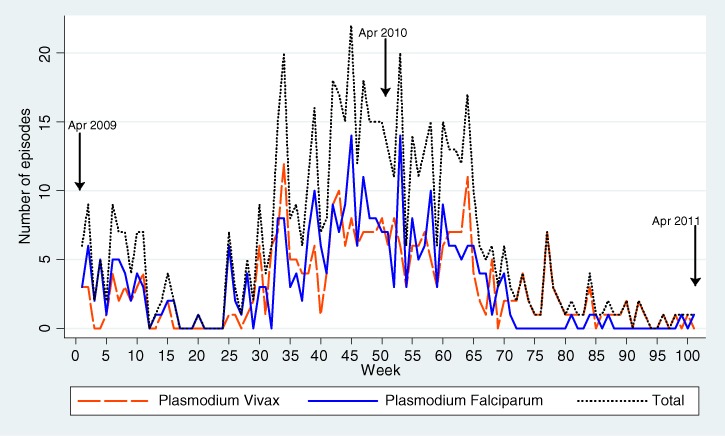
Sequence chart of *Plasmodium falciparum*, *Plasmodium vivax*, and total episodes over 101 weeks of observation, Chano Mille Kebele, south Ethiopia, 2009–2011.

During the 101 weeks of follow-up, there were 2,573 microscopically screened febrile episodes. Of these, 624 (24.3%) were microscopically confirmed malaria episodes; falciparum and vivax malaria accounted for 317 (50.8%) and 307 (49.2%) episodes, respectively. The pattern of malaria occurrence over the whole study period is shown in Figure 3. Descriptive statistics of meteorological variables and malaria episodes are presented in Table 1.

Of 317 falciparum malaria episodes, 224 (70.7%) were among registered residents and the rest 93 (29.3%) were temporary residents or visitors. Among 93 temporary visitor cases, 67 were males; their mean (SD) age was 14.8 (6.9) years. Only 12 of 93 cases were later registered in the last two censuses. Qualitative inquiry showed that most of the temporary residents or visitors were labor migrants such as house servants and cattle shepherds from neighboring districts and zones. Characteristics of registered residents by number of falciparum malaria episodes are presented in Table 2.

The incidence of falciparum malaria among registered subjects was 3.57/10,000 person-weeks of observation. As compared with females, males had a 64% higher rate of acquiring falciparum malaria. Likewise, the rate for the poor was greater than the rich by 64%. Among all age categories, children 5–14 years of age had higher IRR. The IRR was less for sub-Kebele*s* 1 and 2, and for the weeks in which the total rainfall was < 1.5 mm. Higher IRR was also documented in the first year of the study and after free mass ITNs distribution but before IRS with Deltamethrin. There was no significant difference in IRRs among different levels of education of the household head (Table 3).

We also calculated the IRRs by stratifying the ITNs use fraction into different age categories. This showed that before mass ITNs distribution, children 5–14 years had significantly higher IRR followed by under five children, but there was no significant difference between age categories 15–24 and above 24 years. After free mass ITNs distribution, the IRRs of < 5 and 5–14 years categories declined and there was a significant increase in IRR of 15–24 years category, whereas the IRR of 5–14 years category was still the highest. Meanwhile, after IRS with Deltamethrin (ITNs use fraction of 0.63), no significant differences were observed in the IRRs of all age categories (Table 4).

Regarding malaria-related interventions, IRS was carried out in June 2009 (DDT)—Week 7 and July 2010 (Deltamethrin)—Week 63; and free ITNs were distributed to all households in March 2010—Week 48. Post-intervention surveys showed that the coverage of IRS with DDT and Deltamethrin was 91% and 97.5%, respectively. The percentages of households that practiced replastering of the sprayed surfaces was 9.2 (DDT) and 3.2 (Deltamethrin). Meanwhile, an average of 2.3 ITNs were given to each household (a household had an average of 5.9 persons).

### Predictors of weekly number of falciparum malaria episodes.

**Figure 4.**
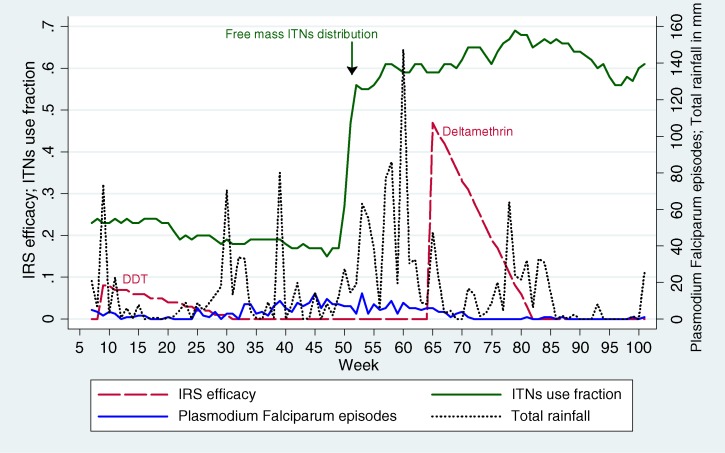
Sequence chart of *Plasmodium falciparum* episodes, total rainfall (lagged by 6 weeks), indoor residual spraying (IRS) efficacy (lagged by 2 weeks), and insecticide-treated nets (ITNs) use fraction (lagged by 2 weeks), Chano Mille Kebele, south Ethiopia, 2009–2011.

**Figure 5.**
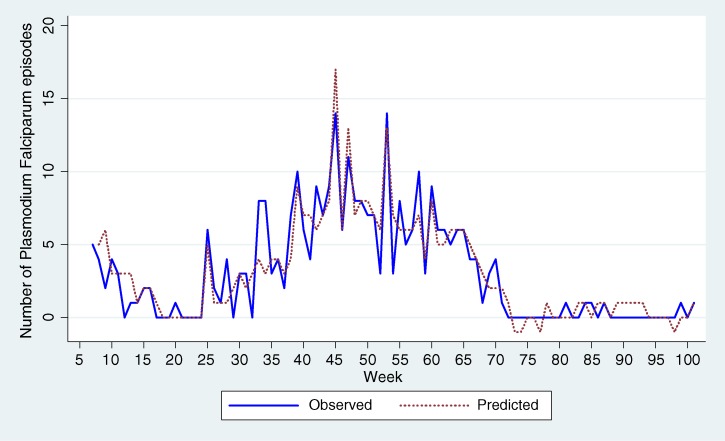
Actual and model-predicted *Plasmodium falciparum* episodes over 95 weeks of observation, Chano Mille Kebele, south Ethiopia, 2009–2011.

Time series modeling with a serial length of 95 weeks was carried out. The total number of falciparum malaria episodes from Weeks 7 to 101 was 295 including cases that did not exist in the census register. The average number of episodes per week was 3.1. The minimum, maximum, and mean ITNs use fraction after mass distribution was 0.47, 0.69, and 0.61; before mass distribution, these figures were 0.16, 0.24, and 0.2, respectively. The meteorological variable with a significant peak from the cross-correlation function plot was rainfall (lag number 6). Minimum and maximum temperature and relative humidity had no significant peaks to consider. The ITNs use fraction and IRS with DDT and Deltamethrin were lagged by 2 weeks. Total rainfall and IRS with Deltamethrin significantly predicted the weekly number of falciparum malaria episodes. However, ITNs use fraction was not significant; and separate analyses using data before and after mass ITNs distribution produced a similar result. Figure 4 shows the pattern of the variables considered in the final model and the ITNs use fraction. Table 5 shows the time-series model parameter values of the significant predictors with the specified model structure and Figure 5 shows the performance of the model prediction (based on “DDT + Deltamethrin” model).

### Predictors of total number of falciparum malaria episodes per individual.

The total number of study subjects was 8,121, of these 4,227 (52.1%) were males. The mean (SD) age was 20.08 (15.35) years.

From Weeks 7 to 101, there were 204 episodes of falciparum malaria, of which two subjects had three episodes, 11 had two episodes, and the rest had one episode.

The constructed generalized Poisson log-linear model showed that those who were males, youngest (larger coefficient for age 5–14 years category), poorest and lived closer to the vector breeding place being more at risk. Total number of weeks in which study subjects had slept under ITNs and education of the head of the household did not predict total number of falciparum malaria episodes (Table 6). The pairwise multiple comparisons for age showed the absence of a significant difference between age category < 5 and 5–14 or 15–24 years categories. However, significant difference existed between 5–14 and 15–24 years categories.

### Predictors of falciparum malaria infection at individual level: repeated measurements.

The total number of subjects was 8,071 and the number of measurements per subject ranged between 2 and 95. Although taking into account the repeated measurements and the number of subjects, the total sample size became 582,846. This GEE model involved a total of 199 falciparum malaria episodes based on the availability of data on ITNs use for each individual. And to check for consistency in prediction, the variables used to build generalized Poisson log-linear model were considered.

The constructed GEE model showed those who were males, youngest (larger coefficient for 5–14 years category), poorest, and who lived closer to the vector breeding place being more at risk. The ITNs use was protective. Education of the head of the household was not significant (Table 7). The pairwise multiple comparisons for age showed that there was no significant difference among the three younger age categories except between 5–14 and 15–24 years.

## Discussion

The incidence of falciparum malaria was 3.57/10,000 person-weeks of observation. Of all falciparum malaria episodes, 29.1% were among temporary residents or visitors. Total rainfall, IRS with Deltamethrin, nearness to vector breeding place, sex, age, ITNs use at individual level (not at community level), and wealth index were significant predictors of falciparum malaria.

The availability of both Co-Artem and Chloroquine motivated the residents to seek treatment at the health post, and the active search for cases facilitated prompt diagnosis and treatment. The weekly timescale provided adequate serial length for time-series modeling. The effect of local meteorological factors was evaluated controlling for malaria-related interventions. Different statistical modeling strategies allowed better scrutiny of the role of potential predictors of falciparum malaria. In addition, this study evaluated the effect of ITNs use at an individual level rather than the number of ITNs distributed. However, the data on ITNs use was based on self-report and was collected once in a week. Meanwhile, the population movement was more than expected, and there was no mechanism in place to register newcomers the moment after they arrived apart from the censuses conducted in the middle and at the end. Therefore, some study subjects could not be followed for some of the time they stayed in the study area.

The significant impact of rainfall at a lag of 6 weeks (“shorter lag”) and the absence of effect of other meteorological variables were consistent with the findings of a study conducted in some districts of Ethiopia with comparable temperature and timescale.^6^ The reason behind the analysis of local meteorological conditions in this work was to take into account the confounding effect while making conclusions about the impact of malaria prevention and control measures.

The ITNs use fraction and incidence of falciparum malaria increased in the period between mass ITNs distribution and IRS with Deltamethrin. We suggest that the episodes of falciparum malaria were dependent on other factors like rainfall rather than the ITNs use. Time series modeling showed that ITNs use fraction did not reduce incidence of falciparum malaria. This could also be explained by qualitative evaluation of Figure 4. After free mass ITNs distribution, the increase in ITNs use did not reduce falciparum malaria before IRS with Deltamethrin was introduced. Fifteen weeks is probably long enough to evaluate the impact of ITNs use fraction. Although the proportion of use by adults and children reached 35–65%, a threshold recommended,^19^ the observed ITNs use might not be sufficient to reduce falciparum malaria. A previous study also showed that high ITNs ownership alone did not reduce falciparum malaria.^23^

The generalized Poisson log-linear model also showed that the study subjects were at risk of getting malaria infection regardless of the number of nights they had spent under the ITNs. This model did not consider the lag effect of ITNs use because the total number of nights spent under ITNs was obtained by cumulating the number of times that individuals slept under ITNs and hence, could not guarantee protection against falciparum malaria unless the ITNs were used consistently. Therefore, the absence of a significant effect of total number of nights spent under ITNs on episodes of falciparum malaria might be related to inconsistent or improper use of ITNs.^33^ Though both time-series and generalized Poisson log-linear modeling showed some valuable information about ITNs on the general picture of malaria epidemiology, both modeling approaches used a crude way of assessing link between ITNs and falciparum malaria infection. To compensate this, we used modeling allowing for repeated measurements and lagged effects, and it confirmed ITNs use at individual level as protective. Therefore, this study confirms that ITNs were protective at the individual level but did not show community-wide benefit with the observed ITNs use fraction. The absence of community-wide benefit suggests the excito-repellent effect of ITNs might have outweighed the insecticidal effect. This is because it is the insecticidal effect of the ITNs that results in community-wide benefit, whereas the excito-repellent effect increases the risk of infection among non-users.^34^,^35^

The observed data of falciparum malaria episodes and the simulated efficacy decay of DDT and Deltamethrin showed that IRS with DDT did not reduce falciparum malaria episodes, but IRS with Deltamethrin did. This suggests the community-wide benefit of the IRS with Deltamethrin surpassed that of the ITNs (PermaNet2.0^36^) though the ITNs were coated with the same insecticide.

The greater risk among children 5–14 years of age and male subjects was comparable to other findings.^13^,^37^ In general, younger study subjects had higher incidence rates, and this may be related to a lack of acquired immunity. The free mass ITNs distribution changed the rate of acquiring falciparum malaria among different age categories. Before free mass ITNs distribution IRR was higher in the 5–14 years than among < 5 years of age children. After free mass ITNs distribution, IRR increased among the 15–24 year age group. This may confirm the shift in rate of acquiring falciparum malaria among different age categories after such interventions, as has also been reported by others^26^,^38^,^39^; meanwhile, the absence of a significant difference in the IRRs of all age categories after IRS with Deltamethrin suggests that IRS protects all household members regardless of their age. This may also suggest the differences in IRRs among the age categories before IRS with Deltamethrin being attributable to age-related ITNs usage.

Population movement in a form of labor migration is considered important in malaria^8^ and other vector-born disease^9^ transmissions. This study showed that temporary residents or visitors experienced about a quarter of the number of falciparum malaria episodes. Characterizing these cases was limited because of uncertainties of their denominator. Nevertheless, their malaria risk seems to be higher because their denominator or length of stay is less or shorter than that of the permanent residents. This shows the contribution of population movement to malaria transmission, and its implication in prevention and control, and the need for further study.

In conclusion, there were 3.57 episodes of falciparum malaria per 10,000 person-weeks of observation. The data showed that rainfall at a lag of 6 weeks significantly increased, and IRS with Deltamethrin (but not with DDT) reduced falciparum malaria incidence. The ITNs use fraction did not show community-wide benefit, whereas individual ITNs prevented falciparum malaria in individuals.

## Supplementary Material

Supplemental Tables.

## Figures and Tables

**Table 1:** Descriptive statistics of meteorological variables and malaria episodes on weekly timescale, Chano Mille Kebele, south Ethiopia, 2009–2011

Variables (*N* = 101)	Minimum	Maximum	Mean (SD)
Minimum temperature (°C)	14.2	20.7	18.1 (1.2)
Maximum temperature (°C)	25.1	35.6	30.9 (2.1)
Total rainfall (mm)	0.0	147.5	15.7 (24.0)*
Relative humidity (%)	30.7	75.6	56.1 (11.0)
*Plasmodium falciparum* episodes	0	14	3.1 (3.4)*
*Plasmodium vivax* episodes	0	12	3.0 (2.9)*

* Median: 6.2 (total rainfall), 2 (*Plasmodium falciparum* episodes), and 2 (*Plasmodium vivax* episodes).

**Table 2:** Characteristics of registered residents by number of *Plasmodium falciparum* episodes, Chano Mille Kebele, south Ethiopia, 2009–2011

Variables (*N* = 8,121)		*Plasmodium falciparum* episodes							
		0 (*N* = 7,916)		1 (*N* = 188)		2 (*N* = 15)		3 (*N* = 2)	
		No.	%	No.	%	No.	%	No.	%
Sex	Male	4,100	51.8	115	61.2	10	66.7	2	100.0
	Female	3,816	48.2	73	38.8	5	33.3	0	0
Age in years*	< 5	1,033	13.0	30	16.0	3	20.0	1	50.0
	5–14	2,075	26.2	91	48.4	8	53.3	1	50.0
	15–24	2,279	28.8	38	20.2	4	26.7	0	0
	> 24	2,529	31.9	29	15.4	0	0	0	0
Wealth index: mean (SD)		0.24 (0.98)		0.06 (0.89)		−0.28 (0.85)		−0.68 (1.49)	
Distance (km) from vector breeding place: mean (SD)		2.49 (0.34)		2.22 (0.37)		2.09 (0.39)		1.95 (0.21)	
ITNs used (total number of weeks): Median		30		39		40		63	

* Mean (SD) for 0, 1, 2, and 3 episodes: 20.25 (15.41), 13.94 (11.29), 10.13 (5.57), and 4.79 (5.95) years, respectively.

**Table 3:** Characteristics of the study subjects, study year, total rainfall, and ITNs use fraction by incidence rate ratio (IRR) of *Plasmodium falciparum* malaria, Chano Mille Kebele, south Ethiopia, 2009–2011*

Variables		Person-weeks of observation	*Plasmodium falciparum*		
			Number of episodes	Weekly incidence per 10,000	IRR (95% CI)
Sex	Male	319,559	141	4.41	1.64 (1.25–2.14)
	Female	307,613	83	2.7	1
Age in years	< 5	88,556	39	4.4	3.23 (2.05–5.08)
	5–14	182,875	110	6.02	4.41 (3.03–6.4)
	15–24	143,306	46	3.21	2.35 (1.5–3.69)
	> 24	212,435	29	1.37	1
Sub-Kebele	1	210,396	36	1.71	0.23 (0.17–0.33)
	2	205,938	34	1.65	0.23 (0.16–0.32)
	3	210,838	154	7.30	1
Wealth status	Poor	209,155	86	4.11	1.64 (1.14–2.37)
	Medium	250,294	96	3.84	1.53 (1.07–2.2)
	Rich	167,723	42	2.50	1
Education: head of household	No education	331,838	112	3.38	2.44 (0.36–16.41)
	Primary	159,625	65	4.07	2.95 (0.45–19.35)
	Secondary	128,474	46	3.58	2.59 (0.38–17.46)
	Above secondary	7,235	1	1.38	1
Study year	1	328,656	142	4.32	1.57 (1.2–2.06)
	2	298,516	82	2.75	1
Total rainfall in mm†	< 1.5	207,491	44	2.12	0.46 (0.33–0.66)
	1.5–14.6	208,004	83	3.99	0.87 (0.65–1.17)
	> 14.6	211,677	97	4.58	1
ITNs use fraction (average)	0.20‡	269,935	106	3.93	2.76 (1.9–4.02)
	0.56§	97,011	71	7.32	5.15 (3.56–7.45)
	0.63¶	232,102	33	1.42	1
All subjects		627,172	224	3.57	NA

* ITNs = insecticide-treated nets; CI = confidence interval; NA = not applicable.

† Divided into tertiles.

‡ Before free mass ITNs distribution (Weeks 5–47); data on ITNs use was not collected for Weeks 1–4.

§ After free mass ITNs distribution and before indoor residual spraying (IRS) with Deltamethrin (Weeks 48–62).

¶ After IRS with Deltamethrin (Weeks 63–101).

**Table 4:** ITNs use fraction and age categories by incidence rate ratio (IRR) of *Plasmodium falciparum* malaria, Chano Mille Kebele, south Ethiopia, 2009–2011*

Variables		Person-weeks of observation	*Plasmodium falciparum*		
ITNs use fraction (mean)	Age in years		Number of episodes	Weekly incidence per 10,000	IRR (95% CI)
0.2†	< 5	34,432	21	6.10	4.3 (2.28–8.11)
	5–14	77,904	59	7.57	5.34 (3.13–9.13)
	15–24	65,881	13	1.97	1.39 (0.65–2.99)
	> 24	91,718	13	1.42	1

0.56‡	< 5	14,321	11	7.68	2.75 (1.18–6.4)
	5–14	28,358	31	10.93	3.92 (1.97–7.79)
	15–24	22,090	20	9.05	3.24 (1.54–6.82)
	> 24	32,242	9	2.79	1

0.63§	< 5	36,475	5	1.37	1.81 (0.56–5.83)
	5–14	68,863	13	1.89	2.49 (0.98–6.35)
	15–24	47,525	9	1.89	2.5 (0.92–6.78)
	> 24	79,239	6	0.76	1

* ITNs = insecticide-treated nets; CI = confidence interval.

† Before free mass ITNs distribution (Week 5–47); data on ITNs use was not collected for Weeks 1–4.

‡ After free mass ITNs distribution and before IRS with Deltamethrin (Weeks 48–62).

§ After IRS with Deltamethrin (Weeks 63–101).

**Table 5:** Time series modeling of weekly number of *Plasmodium falciparum* episodes, Chano Mille Kebele, south Ethiopia, 2009–2011*

Model structure	Variables†				Estimate	*P*
ARIMA (0, 1, 5) (0, 0, 0)	Number of *Plasmodium falciparum* episodes		*q*	1	0.887	< 0.001
				5	−0.401	0.022
	Total rainfall (lagged by 6 weeks)‡		TF order	None	0.022	0.001
	IRS efficacy (lagged by 2 weeks)	DDT + Deltamethrin§	TF order	None	−1.867	0.001
		Deltamethrin alone¶	TF order	None	−1.400	0.008
		DDT alone∥	TF order	None	−4.326	0.365

* ARIMA = auto-regressive integrated moving average; TF = transfer function; *q* = moving average order (both orders were significant in DDT alone and Deltamethrin alone models); IRS = indoor residual spraying.

† *Plasmodium falciparum* incidence and total rainfall had first order of non-seasonal differencing.

‡ The reported estimate and *P* value in the table was for the model incorporated “DDT + Deltamethrin.” Rainfall was significant while the model incorporated Deltamethrin alone (estimate = 0.022 and *P* = 0.002) and DDT alone (estimate = 0.017 and *P* = 0.017).

§ Ljung-Box Q *P* = 0.747, and Goodness of fit (Stationary R-squared) = 75.2%; “DDT + Deltamethrin” represents the model that incorporated both but does not mean that both were sprayed together or their interaction term has been used.

¶ Ljung-Box Q *P* = 0.282, and Goodness of fit (Stationary R-squared) = 71.8%.

∥ Ljung-Box Q *P* = 0.368, and Goodness of fit (Stationary R-squared) = 70.6%.

**Table 6:** Predictors of total number of *Plasmodium falciparum* episodes per individual: generalized Poisson log-linear model, Chano Mille Kebele, south Ethiopia, 2009–2011

Variable (*N* = 8,121)		Bivariate			Multivariate		
		Coefficient	Wald χ^2^	*P*	Coefficient	Wald χ^2^	*P*
Sex: Male		0.503	11.06	0.001	0.485	10.68	0.001
Age in years*†	< 5	1.215	21.77	< 0.001	1.117	19.47	< 0.001
	5–14	1.463	46.28	< 0.001	1.343	39.98	< 0.001
	15–24	0.742	8.72	0.003	0.747	9.09	0.003
Wealth index		−0.271	11.91	0.001	−0.186	6.49	0.011
Distance (meters) from vector breeding place		−0.002	87.12	< 0.001	−0.002	83.55	< 0.001
ITNs use: total number of weeks an individual slept under ITNs		1.913E-5‡	7.52E-5	0.993	NA		
Education: head of household‡§	No education	0.803	0.648	0.421	NA		
	Primary	0.980	0.957	0.328			
	Secondary	0.916	0.830	0.362			

* Reference category: > 24 years.

† Age as continuous variable had: bivariate (Coefficient = −0.039, Wald χ^2^ = 34.67, and *P* < 0.001) and multivariate (Coefficient = −0.035, Wald χ^2^ = 29.59, and *P* < 0.001).

‡ Omnibus test was not significant (the model did not outperform the intercept-only model).

§ Reference category: above secondary.

ITNs = insecticide-treated nets; NA = not applicable.

**Table 7:** Predictors of *Plasmodium falciparum* malaria episodes: GEE model, Chano Mille Kebele, south Ethiopia, 2009–2011

Variable (*N* = 582,846)		Bivariate			Multivariate		
		Coefficient	Wald χ^2^	*P*	Coefficient	Wald χ^2^	*P*
Sex: male		0.532	11.65	0.001	0.497	10.48	0.001
Age in years*†	< 5	1.184	20.09	< 0.001	1.019	15.64	< 0.001
	5–14	1.417	41.44	< 0.001	1.163	29.25	< 0.001
	15–24	0.782	9.32	0.002	0.644	6.6	0.01
Wealth index		−0.233	8.04	0.005	−0.155	4.11	0.043
Distance (meters) from vector breeding place		−0.002	85.34	< 0.001	−0.002	83.32	< 0.001
ITNs user		−0.584	13.36	< 0.001	−0.496	9.97	0.002
Education: head of household‡	No education	−0.035	0.001	0.972	NA		
	Primary	0.112	0.015	0.903			
	Secondary	−0.019	3.599E-4	0.985			

* Reference category: > 24 years.

† Age as continuous variable had: bivariate (Coefficient = −0.038, Wald χ^2^ = 37.31 and *P* < 0.001) and multivariate (Coefficient = −0.032, Wald χ^2^ = 25.31 and *P* < 0.001).

‡ Reference category: above secondary.

GEE = generalized estimating equation; ITNs = insecticide-treated nets; NA = not applicable.
