# Supplementary material for: Predictors of Plasmodium falciparum Malaria Incidence in Chano Mille, South Ethiopia: A Longitudinal Study
Source: Am J Trop Med Hyg. 2012 Sep 5;87(3):450–9. doi: 10.4269/ajtmh.2012.12-0155 (PMC3435347; doi:10.4269/ajtmh.2012.12-0155)
Supplement: Supplemental Tables. [file SD1.pdf]

CONSTRUCTION OF WEALTH INDEX

Principal component analysis (PCA) was carried out using 15 variables. Eleven of the variables were binary and the other four variables were dichotomized into meaningful categories (some of the original categories had very few frequencies (Table SF1). Other socio-economic variables were also analyzed but did result in smaller Eigen values. The total variance explained by the first principal component and the corresponding Eigen value was 20.35% and 3.05, respectively. The inclusion of education of the household head in the PCA reduced the total variance explained by 1%, so we opted not to consider it in the wealth index construction rather to use it separately in further analysis. It is also known that the majority of the household heads depend on farming to generate income rather than being used on the merits of their educational status. Table SF2 shows frequencies, communalities, and correlations.

Supplementary Table SF1

Variables and assigned values

S. no.	Variables	Assigned values
1	Electricity	Present = 1, Absent = 0
2	Watch	Present = 1, Absent = 0
3	Radio	Present = 1, Absent = 0
4	TV	Present = 1, Absent = 0
5	Mobile	Present = 1, Absent = 0
6	Refrigerator	Present = 1, Absent = 0
7	Separate room used for kitchen	Present = 1, Absent = 0
8	Bicycle	Present = 1, Absent = 0
9	Any land used for agriculture	Present = 1, Absent = 0
10	Livestock	Present = 1, Absent = 0
11	Account in bank or credit association	Present = 1, Absent = 0
12	Main material of the floor	Cement/ceramic tiles = 1* Earth or dung = 0
13	Main material of the roof	Corrugated iron or cement/concrete = 1† Thatch or leaf = 0
14	Main material of the wall	Wood with mud/wood with mud and cement = 1‡ No wall§/only wood = 0
15	Latrine facility	Pit latrine¶ = 1, No latrine = 0

*Ceramic tile floor: 2 households.
†Cement/concrete roof: 2 households.
‡Wood with mud and cement: 3 households.
§No wall: 2 households.
¶No other specific types reported.

Supplementary Table SF2

Frequencies of the binary/dichotomized variables, communalities, and correlations with the first component

Variable (N = 1388)	Present or favorable		Communalities*	Correlations with the first component†
	Frequency	Percent		
Electricity	1,166	84.0	0.539	0.602
Watch	722	52.0	0.384	0.579
Radio	873	62.9	0.496	0.644
TV	196	14.1	0.555	0.609
Mobile	223	16.1	0.457	0.497
Refrigerator	15	1.1	0.357	0.245
Separate room used for kitchen	767	55.3	0.393	0.374
Bicycle	255	18.4	0.369	0.538
Any land used for agriculture	743	53.5	0.606	0.396
Livestock	800	57.6	0.576	0.417
Account in bank or credit association	127	9.1	0.568	0.180
Main material of the floor	67	4.8	0.322	0.353
Main material of the roof	913	65.8	0.341	0.541
Main material of the wall	1,354	97.6	0.236	0.185
Latrine facility	1,358	97.8	0.439	0.134

*Estimates of the variance in each variable accounted for by the components (4 components were extracted but we took the first component for further analysis).
†Radio was the most representative (of the first component) and followed by TV, electricity, watch, main material of the roof. . . and latrine facility was the least. The absence of negative values confirms that all variables were positively correlated with wealth (as expected).
